# Etymologia: Usutu Virus

**DOI:** 10.3201/eid2212.ET2212

**Published:** 2016-12

**Keywords:** etymologia, Usutu virus, flavivirus, viruses, Culex neavei, mosquitoes, South Africa, Italy

## Usutu [oo-sooʹtoo] Virus

Usutu virus (Figure), named for the Usutu River in Swaziland, is a mosquitoborne flavivirus closely related to Japanese encephalitis virus, West Nile virus, Murray Valley encephalitis virus, and St. Louis encephalitis virus. Usutu virus was first isolated in 1959 from *Culex neavei* mosquitoes in South Africa. The first recognized infection in a human was in an African man with fever and rash in 1959 but was not reported until 1981.

**Figure:**
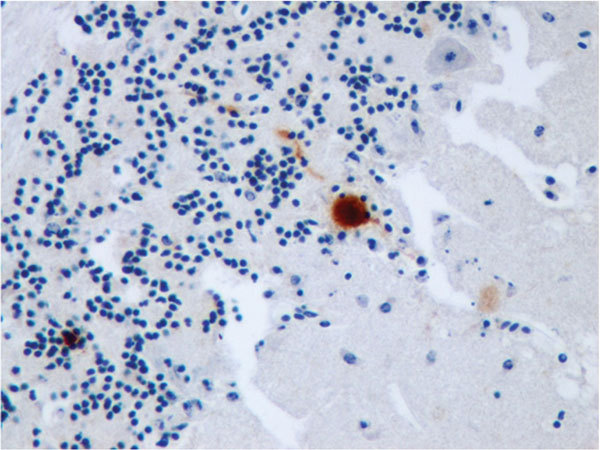
Immunohistochemical staining for Usutu virus antigen in a Purkinje cell of the cerebellum of a song thrush that died of encephalitis. Original magnification ×400.

In 2001, Usutu virus emerged in Europe, when it was identified as the etiologic agent of bird—mainly blackbird—mortality. Retrospective analysis of archived tissue samples from wild bird deaths in the Tuscany region of Italy in 1996, however, revealed an earlier introduction of the virus to Europe. It was not thought to be associated with severe or fatal disease in humans until a neuroinvasive infection was reported to have occurred in an Italian woman in 2009.

## References

[1] Ashraf U, Ye J, Ruan X, Wan S, Zhu B, Cao S. Usutu virus: an emerging flavivirus in Europe. Viruses. 2015;7:219–38. 10.3390/v7010219 25606971 PMC4306835

[2] Pecorari M, Longo G, Gennari W, Grottola A, Sabbatini A, Tagliazucchi S, et al. First human case of Usutu virus neuroinvasive infection, Italy, August-September 2009. Euro Surveill. 2009;14:19446. 20070936

[3] Weissenböck H, Bakonyi T, Rossi G, Mani P, Nowotny N. Usutu virus, Italy, 1996. Emerg Infect Dis. 2013;19:274–7. 10.3201/eid1902.121191 23347844 PMC3559058

[4] Weissenböck H, Kolodziejek J, Url A, Lussy H, Rebel-Bauder B, Nowotny N. Emergence of Usutu virus, an African mosquito-borne flavivirus of the Japanese encephalitis virus group, central Europe. Emerg Infect Dis. 2002;8:652–6. 10.3201/eid0807.020094 12095429 PMC2730324

